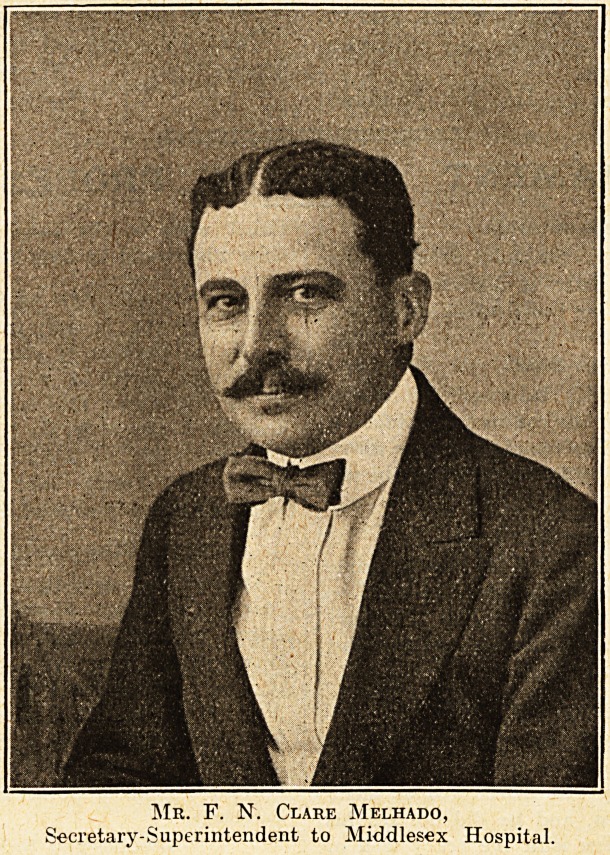# Mr. F. N. Clare Melhado

**Published:** 1916-10-28

**Authors:** 


### MR. F. N. CLARE MELHADO.

### A Valued Hospital Administrator.

By the death of Mr. Clare Melhado, the Middlesex Hospital has sustained a very serious loss. Mr. Melhado was rightly considered by those best able to judge—namely, the officials of the other great general hospitals, as being second to none in his knowledge concerning all that appertains to hospital management. He was appointed clerk in the secretary's office of the Middlesex Hospital, London, on January 14, 1879, and remained in the service of this institution till the day of his death. During this period great changes were made in the management of the hospital and its medical school; the latter was entirely rebuilt, whilst the hospital was considerably altered and improved. In 1885 a new outpatient department was built. In 1888 Mr. Melhado was appointed Secretary-Superintendent; that year saw the completion of the Residential College and the Trained Nurses' Institute. In 1891 the offices of steward and outside collector were abolished, and the whole of the duties of administration were placed under the charge of the Secretary - Superintendent. In 1896 the large convalescent home at Clacton-on-Sea was opened, and in this year the amalgamation of the hospital and medical school was effected. In 1899 the medical school was rebuilt; in 1901 a laundry at Hendon, and in 1902 a nurses' home were erected. In 1909 and 1910, as the result of the Barnato-Joel bequest, the number of beds of the Cancer Charity was increased to ninety-two; magnificent new buildings were erected, including a nursing home, outpatient department, operating theatre, cancer research laboratories, and an extensive department for $x$-rays, radiant heat, and other electrical treatment. In 1914 the Bland-Sutton Institute of Pathology was opened, and in 1915 the children's wards were reconstructed and enlarged. In addition the hospital itself was enlarged by the extension of its two wings, whilst many and great improvements were effected in it in other ways. In all of these additions, alterations, and improvements, Mr. Melhado's was the guiding hand, and whilst most of them owed their initiation to his foresight, the successful establishment of all of them was due in a large measure to his remarkable business capacity and the great industry he exercised on their behalf. His devotion to the hospital was the absorbing passion of his life, and he laboured incessantly on its behalf year in and year out, with but scant periods of relaxation, for just upon thirty-seven years.

Mr. F. N. Clare Melhado,
Secretary-Superintendent to Middlesex Hospital.

Mr. Melhado was especially interested in the hospital chapel, which was erected in 1901 to the memory of the late Chairman, Major A. H. Ross, and it was primarily owing to Mr. Melhado's exertions that the internal decorations, as devised by the architect, Mr. John Loughborough Pearson, which make it the most beautiful chapel of its kind in the United Kingdom, have been completed.

### Appreciation by Chairmen.

Among the Chairmen under whom the late Secretary-Superintendent served may be mentioned the present Chairman, H.S.H. Prince Alexander of Teck, H.S.H. the late Prince Francis of Teck, Lord Sandhurst, Major-General Lord Cheylesmore, Sir Ralph Thompson, and Major A. H. Ross. Prince Alexander of Teck has written to Mrs. Melhado: "Your husband was a most faithful friend and an untiring and energetic servant of the hospital. I deeply mourn his loss, as a friend, and on account of the great services he has rendered to the Middlesex during the many years he worked for its welfare. I had spent the afternoon with him only a fortnight ago when on leave, and his death is a great shock to me." Lord Sandhurst writes: "It is nearly thirty years since I was mainly instrumental in our poor friend becoming secretary. It was impossible to have had a more tactful, efficient, and loyal officer than the weekly board had in Mr. Melhado. And since the days when I was last Chairman I frequently sought his advice, and always profited by it. His loss is a very great one, and I am personally very sorry."

Outside his hospital work, Mr. Melhado had a host of friends, whose experience had taught them the value of his friendship. His readiness to help was typical of the man. He took a great interest in Freemasonry, and was one of the founders of the Middlesex Hospital Lodge, of which he was the Worshipful Master in 1907-1908. He had also twice been Worshipful Master of the Middlesex Lodge, and held Grand rank as Past Assistant Director of Ceremonies.

About 10 P.M., October 17, at the house of one of his greatest friends, Mr. Melhado had an apoplexy. He remained conscious for a short time and died in the early hours of October 18. He was born on February 14, 1862, being the son of Mr. A. Melhado, a merchant of Jamaica, and grandson of Sir Frederick Armstead Steele, Bart. He married Adelaide, the second daughter of the late Mr. Charles Dufresne, of Montreal. It may truthfully be said that no one was ever connected with the Middlesex Hospital who worked harder to ensure its success than the late Secretary-Superintendent.

### Letter from the Queen.

Her Majesty the Queen has written as follows:—

Buckingham Palace,
21st October, 1916.

Dear Mrs. Melhado,

The Queen desires me to tell you how grieved she is to hear of the sad death of your husband, and to express Her Majesty's heartfelt sympathy with you in your overwhelming sorrow.

In addition to having met the late Mr. Melhado on several occasions, the Queen well remembers the kind and valuable assistance he gave at all times to her two brothers.

Her Majesty feels sure that your husband's death will be universally regretted, and she fully realises what the loss must mean to you.

May I be allowed to add my personal condolences?

Yours sincerely,
(Signed) Edward Wallington.

## Figures and Tables

**Figure f1:**